# Efficacy of pre-operative silodosin on flexible ureteroscopy procedure: A randomized controlled study

**DOI:** 10.1080/2090598X.2023.2208790

**Published:** 2023-05-09

**Authors:** Hussein Shaher, Ahmed Sebaey, Ahmed Mahmoud Abd Albaky, Mahmoud Abd Alazeem Mahmoud, Ashraf Mohammed Abd Elaal

**Affiliations:** Urology Department, Faculty of Medicine, Benha University, Benha, Egypt

**Keywords:** Flexible ureteroscopy, laser lithotripsy, silodosin

## Abstract

**Objectives:**

To evaluate the impact of silodosin on stages of flexible ureteroscopy (F-URS) procedures, complications, and stone-free rate (SFR).

**Patients and Methods:**

A prospective, randomized, controlled comparison research was conducted on 106 patients who were randomly allocated into two groups: the study group (52 patients) received F-URS with preoperative daily uptake of 8 mg silodosin for 10 days, and the control group (54 patients) received F- URS without silodosin uptake. Two patients were lost during the follow up in the study group and four patients were also lost in the controls.

**Results:**

Operative time, application access sheath time (AAST), entrance to ureteric orifice time (ETUOT), and entrance to bladder time (ETBT) were significantly lower in the study group compared to controls. Meanwhile, F-URS time & laser time was higher in the study group compared to controls but without statistically significant difference. Complications were insignificalty different between both studied goups with no impact on SFR.

**Conclusion:**

Before ureteroscopy, silodosin, an adjunctive alpha-blocker therapy, was successful in treating stones resulting in shortening the procedural time, with no impact on SFR or complication rate.

## Introduction

Lifetime prevalence rates for urinary stones vary from 1% to 20%, and about 50% of recurrent stone formers have just one-lifetime recurrence. A recent review of first-time stone formers calculated a recurrence rate of 26% in five years [1]. Since the 1950s, technology for the stone disease has been making us use new devices and change the management algorithm every ten years. After the initial report of flexible ureteroscopy (F-URS) in 1990 by Fuchs and Fuchs it has become a dazzling alternative for kidney calculi treatment that are up to 4 cm in diameter [2]. In recent literature, it is reported to have a 70–90% stone-free rate (SFR) with fewer complications and more tolerability [3]. However, the application of F-URS is a complex multi-step act, and it may be difficult if any of the consecutive actions fails during the operation. For instance, the ureteral orifice may not lead to the entrance and advancement of ureteral access sheath (UAS); urethra, external urethral sphincter, prostate, and bladder neck may cause difficulty in UAS placement; and all these factors could complicate the F-URS procedure together. Since being used to treat urolithiasis, F-URS has proven to have outstanding clinical performance and safety. Endoscopic stone therapy has changed recently as a result of the widespread use of disposable ureteroscopes [4]. α-1-blockers have been proven to be an important part of medical expulsive therapy (MET), especially for distal ureteral stones. They relax the ureteral muscle, inhibit ureteral spasms, and cause dilatation of the ureteral lumen [5]. Silodosin has made the F-URS process easier; it appears to be linked to faster procedure times, higher SFR, fewer incidences of complications, and fewer postoperative analgesic requirements [6]. This research set out to determine the consequences of silodosin on stages of F-URS procedures as the entrance to bladder time (ETBT), entrance to ureteric orifice time (ETUOT), application of access sheath time (AAST), F-URS time and laser lithotripsy time (F-URS + LT) and total operation time (TOT)], complications and SFR. In the case of upper ureteric and renal stones 10–30 mm. This study aimed to evaluate the impact of silodosin on stages of the F-URS procedures, complications, and SFR.

## Materials and methods

A prospective, randomized, controlled comparison research was conducted on 106 patients who were randomly allocated into two groups: the study group (52 patients) received F-URS with preoperative daily uptake of 8 mg silodosin for 10 days, and the control group (54 patients) received F- URS without silodosin uptake. Two patients were lost during the follow up in the study group and four patients were lost the controls. The study was done after being approved by the ethics committee at the faculty of medicine, Benha University (REC-FOMBU), and all the studied participants provided their informed consent.

Inclusion criteria were patients of both sexes>18 years old, suffering from 10–30 mm kidney stones and upper third ureter stones.

Exclusion criteria were patients with bleeding tendency, previous urinary tract tumors or previously receive chemotherapy or radiotherapy, previous prostatic or urethral surgery, preoperative JJ stenting, pregnancy, urethral stricture, neurological disorders and any patient risky for anesthesia according to American Society of Anesthesiologists (ASA) Score (4–6). Randomization: Before the procedure, randomization was conducted utilizing a sealed envelope containing a piece of paper with the following choices: either F-URS with Sildosin or F-URS without Sildosin. The participants were randomly allocated into two equal goups: the study group included patients treated by F-URS with preoperative daily uptake of 8 mg silodosin. Controlled group included patients treated F-URS without silodosin uptake.

Pre-operative assessment: A thorough clinical history and physical exam were conducted for all patients.

Pre-treatment laboratory evaluation includes laboratory investigations: urinalysis, urine culture, hemoglobin (Hgb) and serum creatinine, coagulation profile, and pregnancy test in young women.

Radiological studies: plain X-ray of the kidney, ureter, and bladder (KUB) and Computerized tomography (CT) without contrast on the urinary tract.

All procedures were applied when the urine cultures (UC) were sterile. Patients who had positive UC were treated first. Patients with negative UC had a single-dose intravenous antibiotic with a first-generation cephalosporin or quinolone for operations. Operative technique: While the patient was under spinal or general anesthesia, a lithotomy position was assumed. Cystoscope was carried out to place a guide wire within the pelvi-calyceal system, and then a retrograde study was done then sequentially ureteral dilation by

dilators up to 14 F. A safety guide-wire was implanted, and a 11/13 F UAS was introduced into the proximal ureter through the guide-wire. A single-use digital flexible ureteroscope was used for all surgeries (LithoVue 7.7/9.5 F, Boston Scientific, USA). Stones were treated on-site using laser lithotripsy. To perform lithotripsy, holmium: YAG laser (LumenisR, pulse 30 Hz, Germany) was set at (0.6–1.2 J) for fragmentation with frequency (15-20 Hz) and at (0.2–0.5 J) for dusting with frequency (15–25 Hz), with maximum power (30 W). A nitinol basket was used to remove large fragments. (JJ was inserted after the surgery, as needed) if JJ was not inserted, the ureteric catheter was inserted for 24 to 48 hours following the procedure then was withdrawn, and the stone clearance was evaluated at 24 hours by KUB. All patients were instructed to come for a subsequent evaluation utilizing CT without contrast on the urinary tract 4 weeks later. If there were no major stone fragments (≤4 mm) or no symptoms of hydronephrosis, the patient was deemed stone-free. Complications like fever, hematuria, and mucosal injury were reported and classified according to the Clavien classification.

Data from each group, including age, sex, stone size, operation time, results of moving ureteroscope forward to reach the stones, a requirement for ureteric orifice dilation, complications, and SFR, were logged and analyzed. Post-operative follow-up: The SFR one day by KUB and one month by CT. Primary endpoint: ETUOT was seen by comparing between two groups, and secondary endpoint: SFR was seen by comparing the preoperative and 1-month postoperative CT. Stone-free is characterized as the absence of fragments upon radiologic examination of the KUB 1 day postoperatively and the CT 1 month postoperatively. Non-significant residual stones (NSRS) are those that are less than 4 mm in size and cause no pain. Significant residual stones (SRS) are described as those that are less than 4 mm in size and cause discomfort or those that are greater than 4 mm.

## Sample size and statistical analysis

The Open Epi program version 3 was used to calculate the sample size and in keeping with a prior study conducted by Kopru et al. who claimed in their research that the entrance to bladder time (ETBT) (sec) was found lower in group I (intervention group) (8.84 ± 3.37) than group II (control group) (17.45 ± 12.59) with statistically significant difference between the two groups with a mean difference of 8.61 and with p-value = 0.001; and modifying confidence interval to 95%; power of the test to 90% and the proportion between groups is 1:1; the minimum sample size was found 50 cases (25 cases per group) [7].

Using SPSS 22.0 for Windows, all data were gathered, tabulated, and statistically examined (SPSS Inc., Chicago, IL, USA). Using the Shapiro Walk test, the distribution of data was examined for normality. The qualitative data was exhibited using frequencies and relative percentages. The chi-square test and Fisher exact were utilized to determine the variation between the qualitative variables (2). For parametric and non-parametric data, respectively, mean and SD (standard deviation) were employed to express quantitative data. Independent T-test and Mann-Whitney test were utilized to determine the difference between quantitative variable in the two groups for parametric and non-parametric variables, respectively. P-values≤0.05 were used in all two-tailed statistical comparisons to denote significance.

## Results

**Figure 1.**
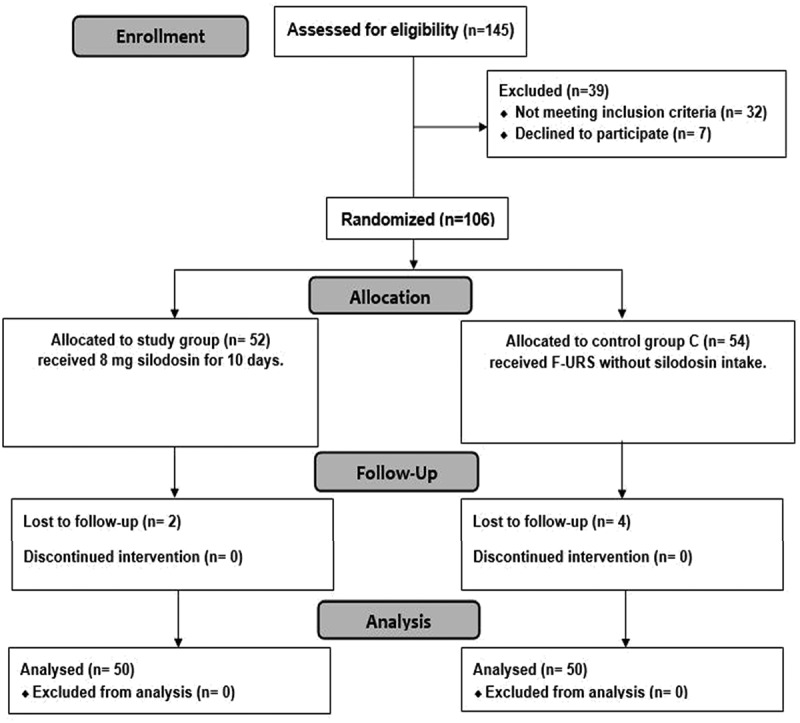
CONSORT flowchart of the studied patients.

In the present study, 145 participants were screened for participation; 32 patients have not met the inclusion requirements, and 7 patients declined to participate. 106 patients were randomly distributed into two treatment groups, 52 patients for the study group and 54 patients for the control group. Only 2 patients were lost in the follow up in the study group and 4 patients were also lost in the controls. 100 patients were monitored and statistical analysis was performed (Figure 1).

There were no significant variations regarding age, gender, BMI, comorbidities, operation side, stone location, stone characteristics, and hydronephrosis between the studied groups (Table 1).

**Table 1.** General characteristics of the examined groups.

	Study (*n* = 50)		Control (*n* = 50)		P
Age (years)	44.65 ± 10.13		45.37 ± 12.78		0.756
**Gender**					
Male	37	(74%)	30	(60%)	0.137
Female	13	(26%)	20	(40%)	
BMI (kg/m^2^)	26.12 ± 2.63		26.34 ± 2.74		0.683
**Comorbidities**					
Diabetes mellitus	9 (18%)		6 (12%)		0.401
Hypertension	6 (12%)		5 (10%)		0.749
Smoking	18 (36%)		17 (34%)		0.834
**Operation side**					
Right	28	(56%)	24	(48%)	0.423
Left	22	(44%)	26	(52%)	
Stone location					
Renal pelvis	21	(42%)	24 (48)		0.929
Upper ureter	11	(22%)	8 (16%)		
Middle calyx	9 (18%)		10	(20%)	
Lower calyx	5 (10%)		5 (10%)		
Upper calyx	4	(8%)	3	(6%)	
**Stone characteristics**					
Density (HU)	837.64 ± 234.3		819.5 ± 240.1		0.703
Size (mm)	18.33 ± 5.17		17.61 ± 4.25		0.449
Volume (mm3) Median (range)	250 (140–721)		242(134–690)		0.699
**Hydronephrosis**					
Grade I	25	(50%)	25	(50%)	0.943
Grade II	19	(38%)	20	(40%)	
Grade III	6 (12%)		5 (10%)		

Data are presented as mean ±SD or number (percentage); BMI, body mass index.

Operative time, AAST, ETUOT, and ETBT were significantly lower in the study groups compared to controls. Meanwhile, F-URS time and laser time was higher in the study group when compared to the controls without statistically significant differences. However, the percentage of JJ stent insertion after operations was comparable in both groups without a statistically significant difference (Table 2). SFR on day one and one-month after was insignificantly different between both studied goups (Table 2).

**Table 2.** Clinical characteristics and outcome of the examined groups.

	Study (*n* = 50)	Control (*n* = 50)	P
Total Operative time (min)	107.43 ± 32.61	178.59 ± 41.54	<0.001*
F-URS time & laser time (min)	61.3 ± 30.47	50.24 ± 25.68	0.053
AAST (min)	18.25 ± 6.29	46.51 ± 15.73	<0.001*
ETUOT (min)	24.58 ± 12.64	86.53 ± 14.9	<0.001*
ETBT (min)	8.43 ± 3.41	18.27 ± 11.52	<0.001*
JJ stent insertion after operations	26 (54%)	30 (60%)	0.421
Stone free rate distribution			
1^st^ day (initial outcome)	35 (70%)	36 (72%)	0.826
One month (late outcome)	42 (84%)	39 (78%)	0.444

ETBT: Entrance to bladder time, ETUOT: Entrance to ureteric orifice time, AAST: Application of access sheath time, *: significant as P-value<0.05.

NSRS on 1st day was 24% and after one month was 12%, and SRS on 1st day was 6% and after one month was 4% in the study group. While NSRS on 1st day was 22% and after one month was 14%, and SRS on 1st day was 6% and after one month was 8% in the control group. However, residual stones were insignificantly different between the studied groups. No significant difference was observed between the studied groups regarding the Clavien classification of complications (*P* = 0.727). In the study group, one patient presented with fever, and 3 patients presented with UTI (grade 1). While in the control group fever was observed in two patients, one patient presented with hematuria, and 2 patients presented with UTI (grade 1). Nevertheless, there was no significant difference in the occurrence of complications between the two groups.

## Discussion

Urolithiasis is a global health issue with growing incidence and prevalence, which is mostly attributable to the rise in type 2 diabetes, obesity, and metabolic syndrome [8–11]. Two studies revealed the efficacy of α-blockers in treating bigger ureteral stones [12,13]. α- blockers are believed to cause relaxation of the ureter’s smooth muscle by blocking its contraction, thereby reducing the frequency and strength of peristalsis [14].

The comparison of clinical characteristics between the groups examined in this study showed that operative time, ETBT, ETUOT, and AAST were significantly lower among the study group as opposed to controls. Meanwhile, F-URS time & laser time were higher among the study group compared to controls but without statistically significant differences. However, the percentage of JJ stent insertion after operations was comparable in both groups without statistically significant differences.

The current study was supported by Kopru et al. analyzed the results of silodosin on stages of the F- URS procedures. The study enrolled 76 patients suffering from 10–30 mm kidney stones. For treatment, patients were randomly split into two groups: The study group had F- URS with preoperative daily uptake of 8 mg silodosin for 10 days, and the control group had F-URS without silodosin uptake. Both groups had similar age, sex, laterality, and stone distribution (location and size). The study revealed that ETBT, ETUOT, and AAST were noticeably lower among the study group as opposed to controls, but the research group’s overall operating time was reduced but without statistical significance. Meanwhile, F-URS time & laser time were higher among the study group as opposed to controls but without statistically significant difference. Also, the percentage of JJ stent insertion after operations was comparable in both groups [7].

Also, Mohey et al. assessed the effectiveness of silodosin therapy on how well huge distal ureteric stones are managed by semi-rigid URS. 127 adult individuals with ureteral stones were incorporated into the research. Patients were split up into two groups at random:

Silodosin group, which consisted of 62 patients, got silodosin (8 mg) for 10 days before URS; the Placebo group, which consisted of 65 patients, received a placebo. There was a good match between the two groups as regard age, sex, BMI, laterality, stone location, density, size, and volume. The study revealed that the Silodosin group had a shorter mean (SD) operative time than the Placebo group, 41.61 (4.67 min) versus 46.85 (4.6 min), respectively [6]. Alaridy et al. provided support for our findings by evaluating and contrasting the therapeutic efficacies of silodosin, Tadalafil, and their combination for the passage of big semi-arid ureteroscopic through the ureteric orifice during ureteral stone therapy. Four categories are established depending on the medication administered in the study, with 136 patients participating: A (silodosin), B (Tadalafil), C (silodosin + Tadalafil), and D. (placebo). The study revealed that the use of silodosin or/and tadalafil decreased operative time significantly, the three groups silodosin, tadalafil, and silodosin + tadalafil were comparable with regard to operative time [15].

Furthermore, Bhattar et al. silodosin and tadalafil were assessed for their safety and effectiveness in terms of making it easier to maneuver a large ureteroscope for the treatment of ureteral stones. 86 individuals with ureteral stones participated in the trial, and they were randomly randomized to receive silodosin, tadalafil, or a placebo before surgery. According to the study, the mean operating times for silodosin, tadalafil, and placebo groups were 35.2 minutes, 34.91 minutes, and 41.14 minutes, respectively. Patients in the silodosin and tadalafil groups needed considerably less time during surgery than those in the control group (*p* = 0.029 and 0.022, respectively), although no differences existed in mean operating time between the two groups (*p* = 0.908) [16].

However, Kim et al. compared the patient’s postoperative results who had fURS with and without preoperative silodosin. The study included 44 and 43 patients in control and study groups, respectively. In contrast to our results, the study reported no significant variation in operative time existed between the studied groups, this may be due to the difference in surgeon experience as well as the procedural instruments [17].

Also, Aydın et al. aimed to measure the efficiency of supplementary silodosin therapy in raising the success rate of semi-rigid ureteroscopy for the removal of ureteral calculi. Patients were split up into three groups at random: those who did not take silodosin (Study group, *n* = 50), those who did so for one day (Control group, *n* = 50), and those who did so for three days (Group 3, *n* = 47). In contrast to our results, the study reported no significant difference between the studied groups relating to the operational time, this may be due to the difference in study settings [18].

There was no discernible distinction between the groups regarding SFR, non-significant residual stones (NSRS), and significant residual stone (SRS) on 1st day and after one month. Agreeing with our results, Kopru et al. revealed that between study groups, there was no significant difference regarding SFR, NSRS, and SRS on 1st day and after 3 months [7]. Also, Mohey et al. revealed that SFR at 48 hours and 4 weeks after surgery was non- significantly higher in the silodosin group as opposed to the control group [6].

However, Aydın et al., revealed that the 3 days use silodosin group had significantly higher 4-week SFR as opposed to both the 1-day use silodosin and placebo groups [18].

In line with the Clavien categorization of complications, there was no significant difference between the study groups in complications following surgery. Moreover, this study exhibited the existence of insignificant variation between the two groups concerning complications including fever, hematuria, and UTI.

Our findings were consistent with Kopru et al., who revealed that no significant distinction existed between silodosin and control groups regarding the Clavien classification of complications [7].

As well, Alaridy et al. stated that no significant distinction existed between the silodosin, Tadalafil, silodosin + Tadalafil, and placebo groups regarding complications including fever, hematuria, and Mucosal injury. Furthermore, they reported that a significantly higher number of patients relating to groups Tadalafil and silodosin + Tadalafil experienced dyspepsia, headache, and backache as opposed to the silodosin and control groups (*P* < 0.05). On contrary, more patients of silodosin and silodosin + Tadalafil groups complained of abnormal ejaculation and dizziness than those of Tadalafil and control groups (*P* > 0.05) [15]. Also, in conformity with our results Kim et al., stated that no significant distinction existed between silodosin and control groups concerning Clavien classification of complications. But in contrast, they reported that Ureteral wall injury and postoperative pain were more frequent in the control group [17].

In contrast to our findings, Mohey et al. found that the placebo group’s complication rate was significantly greater than the Silodosin group’s (20% vs. 6.4%, *P* = 0.036). The disagreement with our results may be due to the difference in sample size and surgeon experience [6].

The short-term follow-up, single stone, one type of laser machine, and one type of F-URS were limitations of this study.

## Conclusions

Before ureteroscopy, silodosin, an adjunctive alpha-blocker therapy, was successful in treating stones resulting in shortening the procedural time, with no impact on SFR or complication rate. Our findings need to be confirmed by more comparative research with larger sample sizes and longer follow-ups to pinpoint risk factors for unfavorable outcomes.

## Abbreviations

BMI: body mass index
F-URS: flexible ureterorenoscopy
BPH: benign prostatic hyperplasia
IRB: international review board
SD: standard deviation
CVT: costoverebral tenderness
Hgb: hemoglobin
UTI: urinary tract infection
KUB: Kidney, ureter and bladder
JJ: double j
CT: computerized tomography
MET: medical expulsive therapy
SFR: Stone free rate
NSRS: non-significant residual stones
SRS: significant residual stone
TOT: total operation time
LT: lithotripsy time
ETBT: Entrance to bladder time
UC: urine cultures
UAS: ureter access sheath
ETUOT: Entrance to ureteric orifice time
AAST: Application of access sheath time.

## Data Availability

Any reasonable request from the appropriate author will result in access to all data.
